# Aiming for the STARs: radiotherapy for ventricular tachycardia—bright future or cosmic gamble?

**DOI:** 10.1093/europace/euae306

**Published:** 2024-12-24

**Authors:** José Luis Merino

**Affiliations:** Arrhythmia and Robotic Electrophysiology Unit, Cardiology Department, La Paz University Hospital, IdiPaz, P. Castellana, 261, 28046 Madrid, Spain

## Abstract

**Graphical Abstract:**
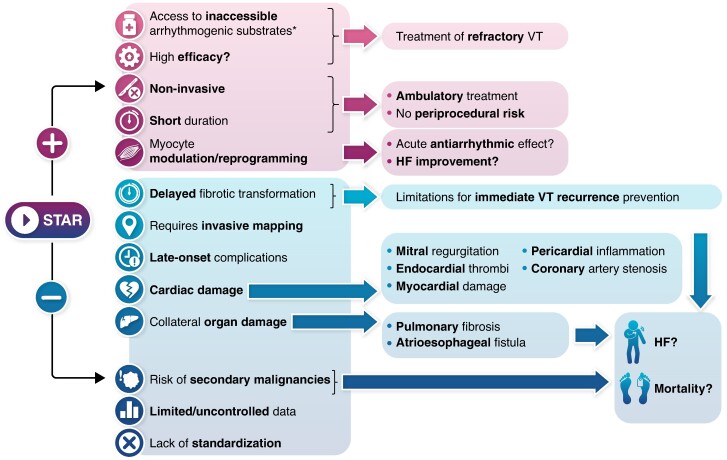

**This editorial refers to ‘Stereotactic cardiac radiotherapy for refractory ventricular tachycardia in structural heart disease patients: a systematic review’ by A. Gupta *et al.*, https://doi.org/10.1093/europace/euae305**.

Ventricular tachycardia (VT) is a life-threatening arrhythmia that can lead to haemodynamic compromise, heart failure, and sudden death. While implantable cardioverter defibrillators (ICDs) effectively terminate VT episodes, frequent ICD shocks are painful and may adversely affect long-term outcomes. Antiarrhythmic drugs help reduce VT burden but have limited efficacy and considerable side effects. Catheter ablation can offer a more definitive approach, yet the VT substrate often lies in mid-myocardial or epicardial regions, making it technically challenging and sometimes requiring epicardial access. Prior cardiac surgeries may result in pericardial adhesions that further complicate the procedure. Additionally, VT ablation carries a significant procedural risk, with major complications reported in up to 6.3%.^1^

A non-invasive therapy that can reduce VT burden without the risks of invasive procedures would be highly attractive. In 2015, Loo *et al*.^2^ introduced the concept of stereotactic arrhythmia radioablation (STAR), and Cuculich *et al*.^3^ popularized it in 2017. Yet, despite promising early results, STAR has not gained widespread adoption. Published data largely consist of case reports and small series.

In this issue of the journal, Gupta *et al*.^4^ present a meta-analysis summarizing much of the existing STAR experience. Given that VT remains difficult to treat and that reducing ICD shocks is always desirable, this contribution merits attention. However, before embracing a new therapy, it is crucial to critically examine the evidence regarding its efficacy, safety, and applicability (*Figure 1*).

**Figure 1:**
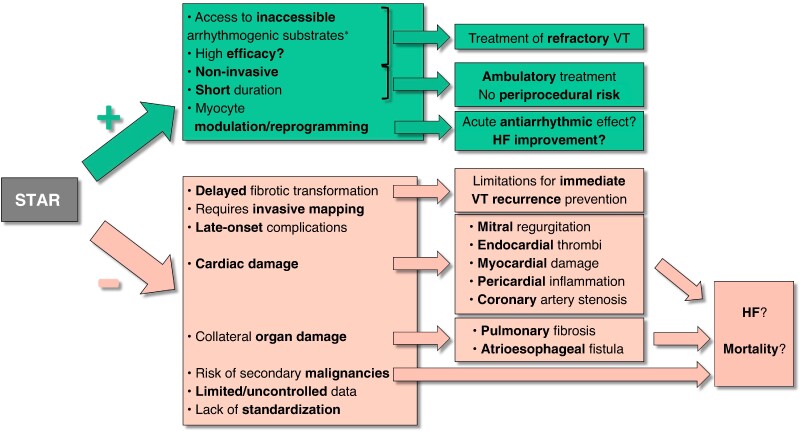
Positive (+) and negative (−) characteristics, potential effects, and consequences of stereotactic arrhythmia radioablation (STAR). HF, heart failure; VT, ventricular tachycardia. *Includes intramural VT substrates, contraindications for endocardial ablation (e.g. LV thrombi), or challenges to endocardial or epicardial ablation, such as valvular prostheses or pericardial adhesions.

## Efficacy considerations

The authors report a 10-fold reduction in VT episodes, a seemingly robust finding. However, several caveats need mentioning. VT often occurs in clusters, with periods of frequent activity followed by long quiescent intervals. Previous data show that after an initial VT storm, only about half of patients have a second storm, and 20% of these occur more than a year after the first event.^5^ This natural fluctuation can create the illusion of therapeutic success if follow-up coincides with a natural ‘cooling off’ period.

In this meta-analysis, a three-month blanking period was employed—VT recurrences during this window were not counted, potentially skewing results. Such an approach may introduce regression to the mean, comparing a peak VT episode period (before therapy) to a naturally quieter phase (after therapy), and thus overestimating efficacy. The three-month blanking also shortened effective follow-up from 13.3 to 10.3 months, complicating comparisons to catheter ablation studies, which rarely use blanking periods. Moreover, 18 patients died during the blanking period, potentially removing patients with a high propensity for VT from later analysis. While the rationale behind a blanking period is the presumed need for STAR-induced fibrotic changes to mature, recent studies suggesting acute changes^6^ and a consensus statement from the European Heart Rhythm Association (EHRA) and the Heart Rhythm Society (HRS) discourage the routine use of a blanking period for STAR.^7^

## Safety concerns

Safety is paramount, especially for a therapy that may offer incremental benefits. Several red flags emerge.

### High mortality and heart failure

The meta-analysis reports a 43% mortality rate at two years. Although patients had advanced heart disease, this figure is substantially higher than those seen in other VT populations, typically under 10% at two years.^8^ This raises the possibility that radiation may contribute to progressive myocardial or collateral damage. The targeted areas averaged ∼3.5 of 17 left ventricle segments—around 20% of the left ventricle mass—potentially compromising a substantial portion of myocardium. Some studies suggest no significant myocardial damage as left ventricular ejection fraction (LVEF) was slightly improved by 1.5%, and others propose a cardiomyocyte ‘conditioning’ phenomenon.^6,9^ However, transient reduction in LVEF due to VT episodes or ICD shocks may recover once these episodes abate, potentially masking subtle radiation damage. Longer follow-up is needed to clarify STAR’s net effect on cardiac function and mortality.

### Atrioesophageal fistula

Two cases of atrioesophageal or pericardioesophageal fistula were reported. Such a complication is essentially unknown in VT catheter ablation and is extremely rare (0.016–0.1%) in atrial fibrillation ablation.^10^ The latency of these complications—9 months to 2 years post-STAR—is particularly concerning. Radioablation may cause late-appearing tissue injury not captured by short-term studies.

### Mitral regurgitation and other complications

Mitral regurgitation progression occurred in 4.4% of patients—rarely heard of in VT ablation and far more frequent than in AF ablation. Such changes likely reflect radiation-induced fibrotic remodelling of the mitral apparatus, potentially worsening heart failure. Other acute injuries (pneumonitis, pericarditis) and late complications (fibrosis, vascular damage, potential malignancies) may also emerge over time. With a mean follow-up of around one year, the current data may underestimate long-term risks. The future incidence of secondary malignancies, while less critical in patients with poor prognosis, should not be dismissed if STAR use expands to broader populations.

## Applicability and adoption challenges

Despite early enthusiasm, fewer than 300 STAR cases have been reported worldwide. Reasons may include as follows.

### Mapping limitations

Stereotactic arrhythmia radioablation ideally requires precise arrhythmia localization to target the critical VT isthmus without harming surrounding tissue. While non-invasive mapping technology has improved, it still cannot consistently match the precision of invasive mapping.^11^ Often, failure of catheter ablation stems from incomplete substrate characterization rather than technical inaccessibility.^12^ This limitation reduces STAR’s utility to a ‘bailout’ after ablation failure due to the latter.

### Procedural complexity

Although non-invasive in delivery, STAR often requires at least some form of mapping—be it non-invasive body surface mapping or data from previous invasive procedures—to identify the target. Thus, the theoretical advantage of a completely non-invasive therapy is limited in practice.

## Future directions

Given the current evidence, STAR remains experimental and best reserved for carefully selected cases where other therapies have failed or are not feasible.^13^ Controlled studies with longer follow-up, such as the ongoing RADIATE-VT trial, are needed to better understand the long-term safety profile and to determine which patients may benefit most.

While STAR brings exciting possibilities as a novel, non-invasive modality for treating VT, we must acknowledge the limitations of the current evidence. The potential for significant and sometimes delayed complications requires prolonged surveillance, and the true efficacy remains uncertain due to methodological factors like blanking periods and regression to the mean. As our understanding evolves, careful, evidence-based patient selection and long-term follow-up will be essential before STAR can earn its place alongside established therapies.

## Data Availability

No new data were generated or analysed in support of this research.

## References

[1] Bazan V, Arana E, Rubio-Campal JM, Calvo D; collaborators of the Spanish catheter ablation registry; Álvarez Acosta L et al Spanish catheter ablation registry. 23rd official report of the Heart Rhythm Association of the Spanish Society of Cardiology (2023). Rev Esp Cardiol (Engl Ed) 2024;77:1026–36. 39313188 10.1016/j.rec.2024.07.014

[2] Loo BW Jr, Soltys SG, Wang L, Lo A, Fahimian BP, Iagaru A et al Stereotactic ablative radiotherapy for the treatment of refractory cardiac ventricular arrhythmia. Circ Arrhythm Electrophysiol 2015;8:748–50. 26082532 10.1161/CIRCEP.115.002765

[3] Cuculich PS, Schill MR, Kashani R, Mutic S, Lang A, Cooper D et al Noninvasive cardiac radiation for ablation of ventricular tachycardia. N Engl J Med 2017;377:2325–36. 29236642 10.1056/NEJMoa1613773 PMC5764179

[4] Gupta A, Sattar Z, Chaaban N, Ranka S, Carlson C, Sami F et al Stereotactic cardiac radiotherapy for refractory ventricular tachycardia in structural heart disease patients: a systematic review. Europace 2024. 2024. 10.1093/europace/euae305 PMC11780863 39716963

[5] Streitner F, Kuschyk J, Veltmann C, Mahl E, Dietrich C, Schimpf R et al Predictors of electrical storm recurrences in patients with implantable cardioverter-defibrillators. Europace 2011;13:668–74. 21156679 10.1093/europace/euq428

[6] Mehrhof F, Hüttemeister J, Tanacli R, Bock M, Bögner M, Schoenrath F et al Cardiac radiotherapy transiently alters left ventricular electrical properties and induces cardiomyocyte-specific ventricular substrate changes in heart failure. Europace 2023;26:euae005. 38193546 10.1093/europace/euae005 PMC10803027

[7] Zeppenfeld K, Rademaker R, Al-Ahmad A, Carbucicchio C, De Chillou C, Ebert M, et al Patient selection, ventricular tachycardia substrate delineation and data transfer for stereotactic arrhythmia radioablation. A clinical consensus statement of the European Heart Rhythm Association (EHRA) of the ESC and the Heart Rhythm Society (HRS). Europace 2024: euae214. doi:10.1093/europace/euae214 PMC12041921 39177652

[8] Arenal Á, Ávila P, Jiménez-Candil J, Tercedor L, Calvo D, Arribas F et al Substrate ablation vs antiarrhythmic drug therapy for symptomatic ventricular tachycardia. J Am Coll Cardiol 2022;79:1441–53. 35422240 10.1016/j.jacc.2022.01.050

[9] Zhang DM, Navara R, Yin T, Szymanski J, Goldsztejn U, Kenkel C. Cardiac radiotherapy induces electrical conduction reprogramming in the absence of transmural fibrosis. Nat Commun 2021;12:5558. 34561429 10.1038/s41467-021-25730-0 PMC8463558

[10] Tilz RR, Schmidt V, Püererfellner H, Maury P, Chun KRJ, Martinek M, et al A worldwide survey on incidence, management, and prognosis of oesophageal fistula formation following atrial fibrillation catheter ablation: the POTTER-AF study. Eur Heart J 2023;44:2458–69. 37062040 10.1093/eurheartj/ehad250 PMC10344651

[11] Abdel-Kafi S, Sramko M, Omara S, de Riva M, Cvek J, Peichl P et al Accuracy of electroanatomical mapping-guided cardiac radiotherapy for ventricular tachycardia: pitfalls and solutions. Europace 2021;23:1989–97. 34524422 10.1093/europace/euab195

[12] Herrera Siklody C, Schiappacasse L, Jumeau R, Reichlin T, Saguner AM, Andratschke N et al Recurrences of ventricular tachycardia after stereotactic arrhythmia radioablation arise outside the treated volume: analysis of the Swiss cohort. Europace 2023;25:euad268. 37695314 10.1093/europace/euad268 PMC10551232

[13] Grehn M, Mandija S, Miszczyk M, Krug D, Tomasik B, Stickney KE et al STereotactic Arrhythmia Radioablation (STAR): the standardized treatment and outcome platform for stereotactic therapy of re-entrant tachycardia by a multidisciplinary consortium (STOPSTORM.eu) and review of current patterns of STAR practice in Europe. Europace 2023;25:1284–95. 36879464 10.1093/europace/euac238 PMC10105846

